# Supplementary material for: Non-STEMI vs. STEMI Cardiogenic Shock: Clinical Profile and Long-Term Outcomes
Source: J Clin Med. 2022 Jun 20;11(12):3558. doi: 10.3390/jcm11123558 (PMC9224589; doi:10.3390/jcm11123558)
Supplement: Supplementary file 1 [file jcm-11-03558-s001.zip › jcm-1772163-supplementary.pdf]

Supplementary Table S1. Pharmacological treatment at discharge.

	All patients (n=101)	STEMI patients (n=70)	NSTEMI patients (n=31)	P value
Aspirin	97 (96.0)	66 (94.2)	31 (100)	0.309
P2Y12 inhibitors	84 (83.1)	55 (78.5)	29 (93.5)	0.085
Clopidogrel	64 (63.3)	39 (55.7)	25 (80.6)	0.016
Prasugrel	16 (15.8)	14 (20.0)	2 (6.4)	0.138
Ticagrelor	4 (3.9)	2 (2.8)	2 (6.4)	0.584
Anticoagulants	14 (13.8)	10 (14.2)	4 (12.9)	1
β-Blockers	82 (81.1)	58 (82.8)	24 (77.4)	0.519
Ivabadine	28 (27.7)	20 (28.5)	8 (25.8)	0.775
ACEIs/ARBs	79 (78.2)	57 (81.4)	22 (70.9)	0.24
MRAs	41 (40.5)	30 (42.8)	11 (35.4)	0.486
Diuretics	67 (66.3)	45 (64.2)	22 (70.9)	0.512
Digoxin	10 (9.9)	7 (10.0)	3 (9.6)	1
Nitrates	24 (23.7)	12 (17.1)	12 (38.7)	0.019
Calcium channel blockers	8 (7.9)	4 (5.7)	4 (12.9)	0.245
Amiodarone	12 (11.8)	8 (11.4)	4 (12.9)	1
Statins	89 (88.1)	61 (87.1)	28 (90.3)	0.649

Values are given as n (%). ACEI, angiotensin-converting enzyme inhibitor; ARB, angiotensin receptor blocker; MRA, mineralocorticoid receptor antagonist.

Supplementary Table S2. Cause of death among hospital survivors who died in the first 5 years of follow-up.

	All patients (n=35)	STEMI patients (n=19)	NSTEMI patients (n=16)	P value
Non-cardiovascular	16 (45.7)	10 (52.6)	6 (37.5)	0.371
Heart failure	8 (22.9)	5 (26.3)	3 (18.8)	0.700
Sudden death	6 (17.1)	3 (15.8)	3 (18.8)	1
Acute myocardial infarction	2 (5.7)	0	2 (12.5)	0.202
Stroke	1 (2.9)	1 (5.3)	0	1
Unknown	2 (5.7)	0	2 (12.5)	0.202

Values are given as n (%).

Supplementary Table S3. Cause of the first cardiovascular readmission in the first 5 years of follow-up among hospital survivors.

	All patients (n=32)	STEMI patients (n=20)	NSTEMI patients (n=12)	P value
Heart failure	22 (68.8)	15 (75.0)	7 (58.3)	0.325
Acute coronary syndrome	6 (18.8)	3 (15.0)	3 (25.0)	0.647
Acute myocardial infarction	3 (50.0)	1 (33.3)	2 (66.7)	1
Unstable angina	3 (50.0)	2 (66.7)	1 (33.3)	1
Stroke	2 (6.2)	1 (5.0)	1 (8.3)	1
Ventricular tachycardia	1 (3.1)	0	1 (8.3)	0.375
Atrial flutter	1 (3.1)	1 (5.0)	0	1

Values are given as n (%).
